# Correlation between ovarian follicular development and Hippo pathway in polycystic ovary syndrome

**DOI:** 10.1186/s13048-023-01305-z

**Published:** 2024-01-12

**Authors:** Zichao Huang, Tianyue Xu, Chunling Liu, Honghui Wu, Linglin Weng, Jieyu Cai, Na Liang, Hongshan Ge

**Affiliations:** 1 Graduate School, Nanjing University of Chinese Medicine, Nanjing, China; 2 Reproduction Medicine Centre, The Affiliated Taizhou People’s Hospital of Nanjing Medical University, Taizhou, China; 3 Center for Reproductive Medicine, the Second Affiliated Hospital of Soochow University, Suzhou, Jiangsu 215004 China; 4 Graduate School, Dalian Medical University, Liaoning, China

**Keywords:** Polycystic ovary syndrome, Hippo pathway, Follicular development

## Abstract

**Background:**

For women of childbearing age, the biggest problem caused by polycystic ovary syndrome (PCOS) is infertility, which is mainly caused by anovulation, abnormal follicular development, proliferation of small antral follicles, and cystic follicles. The mechanism underlying its occurrence is not clear. The abnormal proliferation and development of follicles in PCOS patients is a complex process, which is affected by many factors. The objective of this study was to investigate the relationship between the Hippo pathway and follicular development in PCOS, and to further explore this relationship by using the YAP inhibitor verteporfin (VP).

**Method:**

30 3-week-old BALB/C female rats were randomly divided into control group (n = 10), DHEA group (n = 10) and DHEA + VP group (n = 10). The morphology of ovary and the degree of follicular development were observed by HE staining, and the expression and location of AMH in ovarian follicles were observed by immunofluorescence. The ovarian reserve function index AMH, cell proliferation index PCNA and the ratio of Hippo pathway related proteins MST, LATS, YAP, P-YAP and P-YAP/YAP were detected by Western blot.

**Results:**

After dividing 30 3-week-old female mice into control, dehydroepiandrosterone (DHEA; model of PCOS), and DHEA + VP groups, we found that the number of small follicles increased in the DHEA group compared to the control group. Additionally, in the DHEA group compared to the control group, anti-müllerian hormone (AMH; ovarian reserve index) increased, proliferating cell nuclear antigen (PCNA; cell proliferation index) decreased, and upstream (MST and LATS) and downstream (YAP and p-YAP) proteins in the Hippo pathway increased, though the p-YAP/YAP ratio decreased. VP ameliorated the increases in AMH, MST, LATS, YAP and p-YAP, but did not ameliorate the decrease in the p-YAP/YAP ratio.

**Conclusions:**

This study indicates that the increased small follicles in the ovaries and changes in ovarian reserve and cell proliferation may be closely related to Hippo pathway activation. This suggests that the Hippo pathway may be an important pathway affecting the proliferation and development of follicles and the occurrence of PCOS.

## Background

Polycystic ovary syndrome (PCOS) is a common disease caused by complex endocrine and metabolic abnormalities in women of reproductive age. It is characterized by hyperandrogenism, ovulatory dysfunction, and the formation of polycystic ovaries. Its prevalence ranges from 8 to 13%. Most women with PCOS have clinical symptoms such as abnormal menstruation, hirsutism, obesity, and infertility, which can affect the physical and mental health of women with PCOS [1–3].

Many studies have shown that the number of antral follicles in patients with PCOS is abnormally increased [4–6]. The activation of the Hippo pathway (which is involved in overcoming the growth control property of normal cells) is related to an increased proliferation of small follicles [7]. In patients with PCOS, the abnormal follicular proliferation may be related to the Hippo pathway.

The Hippo pathway, which is composed of a group of conserved kinases, inhibits tissue overgrowth. In mammals, receptors on the plasma membrane sense growth inhibitory signals from the extracellular environment and then act on the downstream effector protein yes-associated protein 1 (YAP) via a series of phosphorylation events. Phosphorylated YAP then interacts with cytoskeletal proteins and becomes trapped in the cytoplasm, where it can be degraded by proteases. Thus, YAP is unable to enter the nucleus to exercise its transcriptional activation function, thereby regulating organ size.

The Hippo pathway involves a core signaling axis. First, Hippo (Hpo; MTS in mammals) is activated under physiological or non-physiological stress. It phosphorylates and thereby activates the protein kinase Warts (Wts; LATS1/2 in mammals). This results in a series of changes in the downstream signaling pathway, including YAP phosphorylation [8]. LATS can thereby regulate cell differentiation, proliferation, apoptosis, and migration, regulate the transcription and translation of genetic material, and maintain the stability of genetic material.

Our previous research showed that when S1P, a LATS blocker, was administered to 3-day-old female rats, the number of small follicles increased with increased S1P concentrations. However, whether the Hippo pathway is activated or inhibited during the development of PCOS, and whether it is involved in the increase in small follicles in patients with PCOS need to be further studied. Therefore, this study established a mouse model of PCOS to explore the changes in the number of small follicles and Hippo pathway activation in the ovarian tissue of PCOS mice. Changes in follicular development after administration of the YAP inhibitor verteporfin (VP) were also assessed in order to further analyze the relationship between the Hippo pathway and follicular development. This exploration of the pathogenesis of PCOS provides a theoretical and practical basis for developing new PCOS therapies.

## Materials and methods

### Establishment of mouse model of PCOS

Thirty 3-week-old mice were randomly divided into control, dehydroepiandrosterone (DHEA), and DHEA + VP groups (10 mice per group). The control mice were injected with 0.2 ml/day sesame oil for 4 weeks. To establish the PCOS model, the DHEA and DHEA + VP mice were subcutaneously injected with 60 mg/kg/day DHEA (dissolved in sesame oil) for 4 weeks. Lastly, the DHEA + VP mice were intraperitoneally injected with 50 mg/kg/day VP for 1 week starting from 3 weeks after the beginning of the DHEA injections.

### Hematoxylin and eosin (HE) staining of ovarian tissue and follicle analysis

The mice were killed by cervical dislocation. Under an anatomical microscope, the two ovaries were removed, as were the adipose tissue on the surface and the covered capsule. Six ovaries (left or right) from six mice per group were randomly selected. The remaining ovaries were stored at − 80℃. The selected ovaries were fixed in 4% paraformaldehyde, dewaxed, embedded in paraffin, sectioned with a thickness of 5 inches, dewaxed, hydrated, stained with hematoxylin for 5 min, stained with eosin for 2 min, dehydrated, subjected to clearing, prepared using a coverslip, and then observed under a microscope to assess the morphological changes. Using a double blind method, six random HE-stained sections per group were analyzed. The ovarian tissue sections with the largest cross-sectional area of the ovaries were selected, and the numbers of follicles, cystic follicles, and corpus lutea at each level were observed.

### Western blotting

The total protein was extracted from ovarian tissues and the protein concentration was determined. Next, the proteins were subjected to sodium dodecyl sulfate polyacrylamide gel electrophoresis (SDS-PAGE), transferred to a polyvinylidene fluoride (PVDF) membrane, blocked, incubated with primary antibody against anti-müllerian hormone (AMH), proliferating cell nuclear antigen (PCNA), YAP, p-YAP, MST, or LATS1, and incubated with secondary antibody. Images were then obtained for analysis.

### Immunofluorescence assays

To assess AMH expression and localization, ovarian tissue sections were subjected to dewaxing, rehydration, inactivation of endogenous peroxidase, heat-induced antigen retrieval, blocking, incubation with primary antibody against AMH in the dark, incubation with secondary antibody, nuclear staining, and preparation using a coverslip. The slides were then placed in a wet box to avoid light and left at 4℃. The film was completed in 12 h. Immunofluorescence was assessed using X.

### Statistical analysis

SPSS 22.0 software was used for the statistical analysis. The continuous data are expressed as mean ± standard deviation. Independent-samples t tests were used for comparisons between two groups, and one-way analysis of variance was used for comparisons among multiple groups. P < 0.05 was considered statistically significant.

## Results

### Changes in body weight and fat distribution in mice

**Fig. 1:**
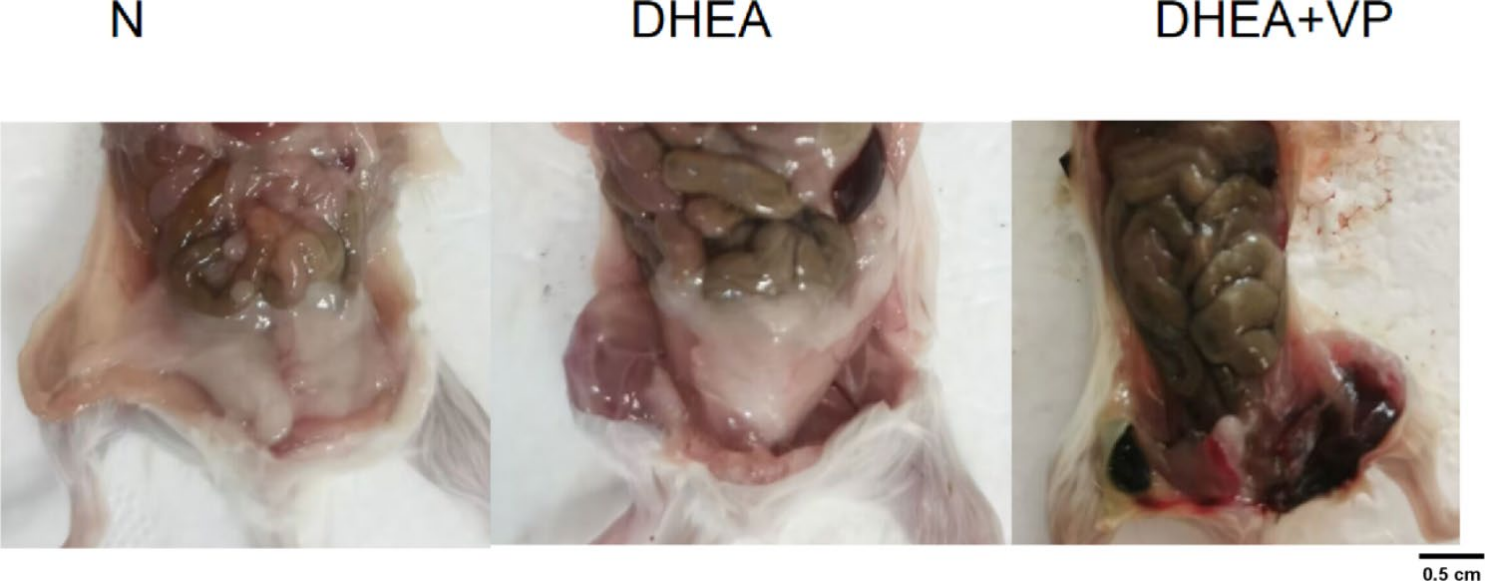
Abdominal fat in the three groups of mice. N: normal control; DHEA: dehydroepiandrosterone; VP: verteporfin

There were no significant differences in body weight among the three groups of mice before the establishment of the PCOS model. At 28 days after the establishment of the PCOS model, the DHEA group showed a slight increase in body weight compared to the control and DHEA + VP groups (Table 1). Dissection showed that the abdominal fat thickness increased in the DHEA group compared to the control group, while the DHEA + VP mice had much less (or even zero) abdominal fat compared to the DHEA group (Fig. 1).

**Table 1:** Body weight of mice before and after establishment of PCOS model

	Before (g)	After (g)
Control group	14.25 ± 0.63	20.93 ± 1.24
DHEA group	13.62 ± 0.41	22.32 ± 1.01
DHEA + VP group	13.56 ± 0.70	21.56 ± 1.55

DHEA: dehydroepiandrosterone; VP: verteporfin. Different superscript letters indicate significant differences (P < 0.05)

### Morphological changes of HE-stained ovarian tissue

**Fig. 2:**
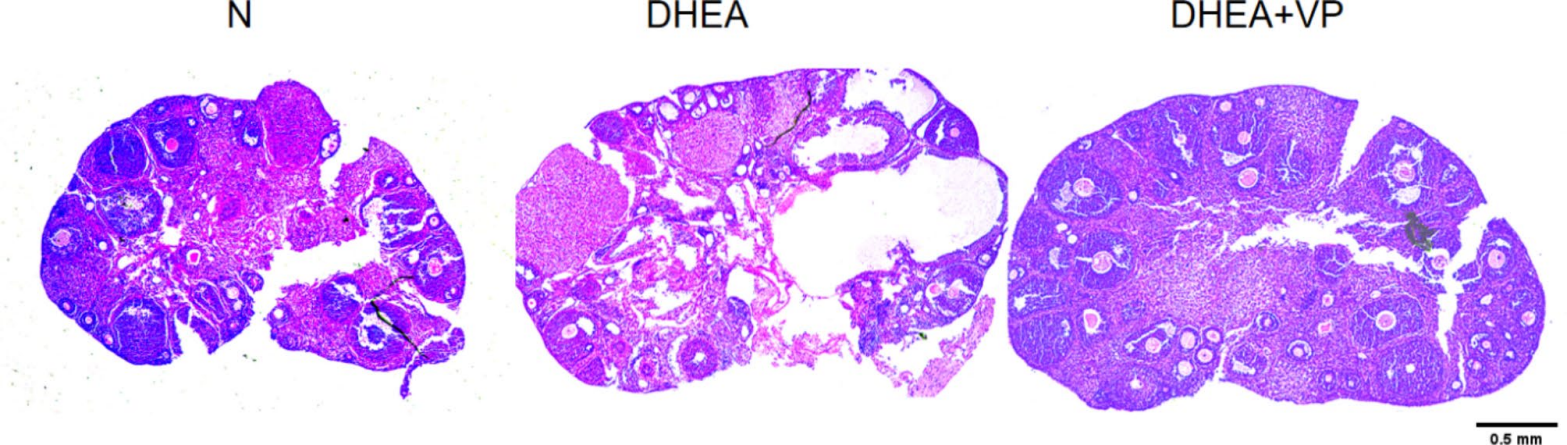
HE-stained ovarian tissues in the three groups of mice (X40). N: normal control; DHEA: dehydroepiandrosterone; VP: verteporfin

The control group had normal ovarian development, based on the size and development of follicles, and all levels of follicular development could be seen. In the DHEA group, the number of small follicles significantly increased and the number of corpus lutea decreased; cystic follicles (with disappearance of the core) appeared (Table 2). This indicated the successful establishment of the PCOS model by subcutaneous injection of DHEA (Fig. 2).

**Table 2:** Follicles and corpus lutea in the three groups of mice

	Control group	DHEA group	DHEA + VP group
Total number of follicles	25.33 ± 2.05	36.67 ± 0.94	36.00 ± 0.82
Growing follicles	20.33 ± 1.70	9.67 ± 1.24	25.33 ± 2.05
Antral follicles	4.33 ± 0.47	25.33 ± 0.94	10.67 ± 2.49
Antral follicle	0.00 ± 0.00	1.67 ± 0.47	0.00 ± 0.00
Corpus luteum	6.33 ± 1.25	2.00 ± 0.82	6.00 ± 1.63

DHEA: dehydroepiandrosterone; VP: verteporfin. Data are based on six ovarian tissue sections per group. The ovarian tissue sections with the largest cross-sectional area of the ovaries were selected. Different superscript letters indicate significant differences (P < 0.05)

### Changes in AMH protein expression in ovarian tissue

**Fig. 3:**
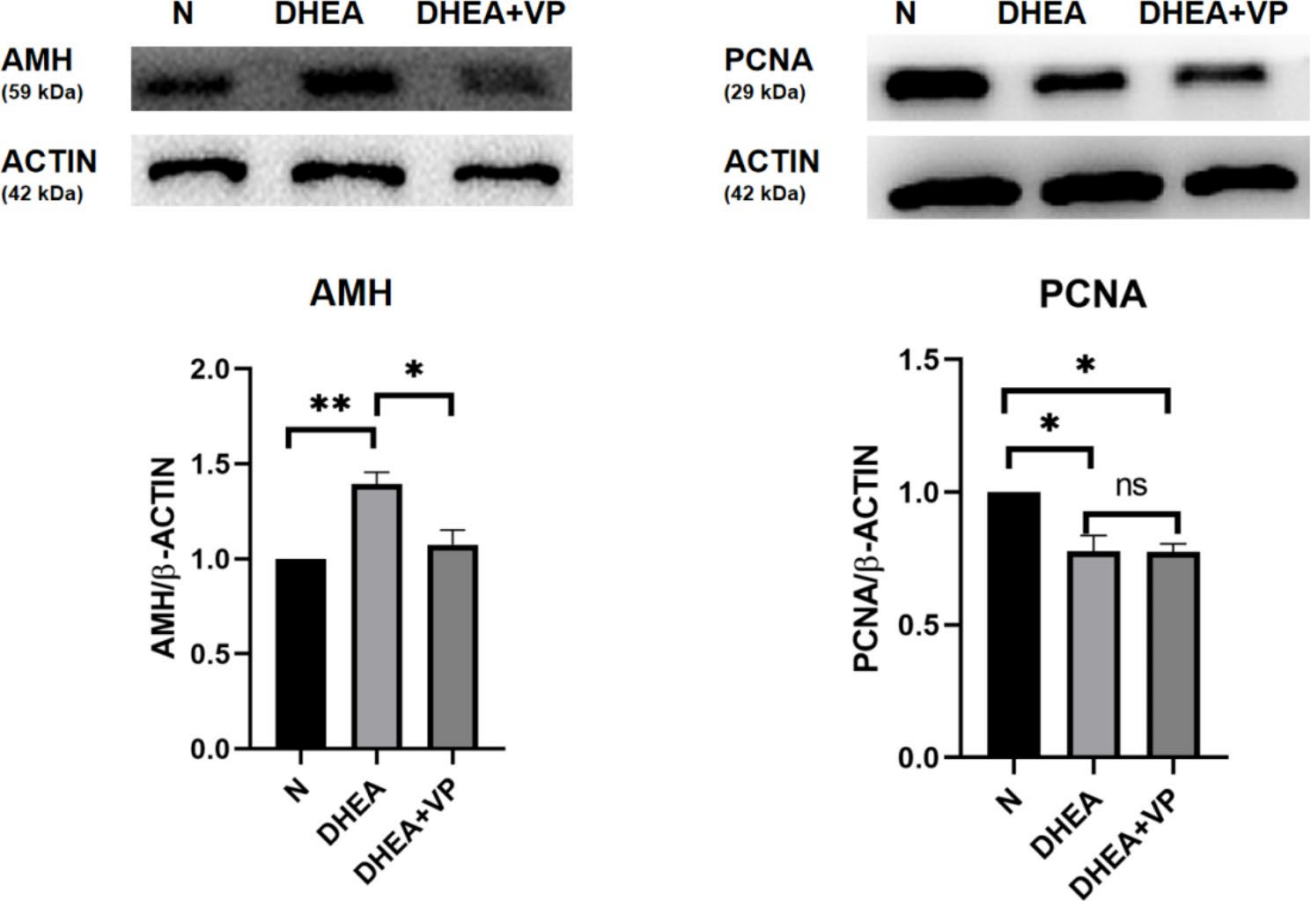
AMH and PCNA protein expression in ovarian tissue in the three groups of mice, assessed by western blotting. N: normal control; DHEA: dehydroepiandrosterone; VP: verteporfin. *P < 0.05, **P < 0.01

**Fig. 4:**
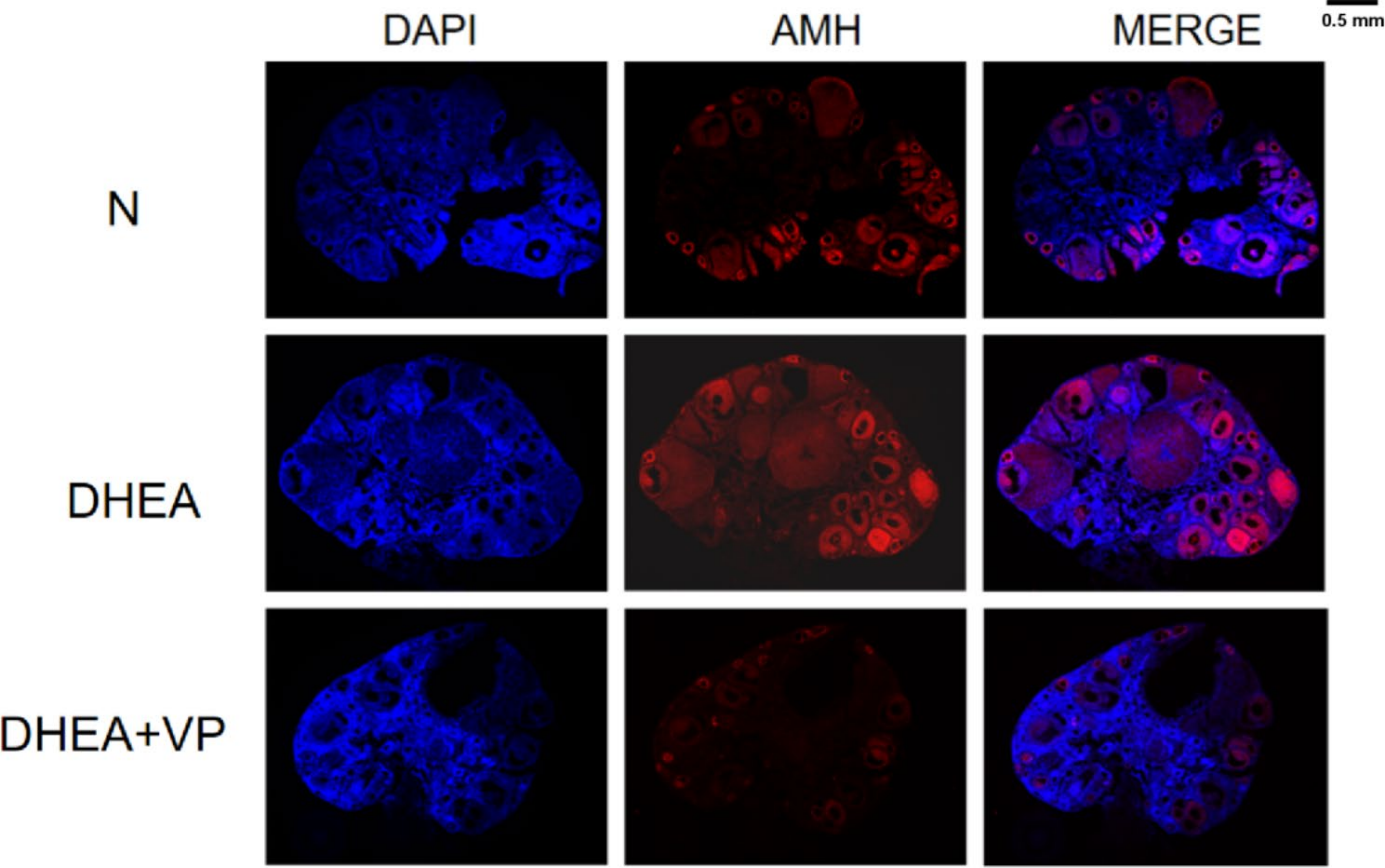
AMH expression and localization, assessed by immunofluorescence assays. N: normal control; DHEA: dehydroepiandrosterone; VP: verteporfin

AMH is a commonly used index to evaluate ovarian reserve. It is mainly secreted by small antral follicles and can be used to evaluate the number of small antral follicles. Western blotting showed that AMH protein expression in ovarian tissue was significantly higher in the DHEA group than the control group, but significantly lower in the DHEA + VP group than the DHEA group (Fig. 3). Immunofluorescence assays showed that AMH was mainly expressed in the granulosa cells of antral follicles (Fig. 4). The number of **antral follicles** was significantly greater in the DHEA group than the control group, but lower in the DHEA + VP group than the DHEA group (Table 2). These results suggest that AMH and the number of **small antral follicles** in PCOS mice increase, while both increases are ameliorated by VP, indicating that VP may inhibit the proliferation of antral follicles in PCOS mice.

### Changes in PCNA protein expression in ovarian tissue

PCNA is related to cell DNA synthesis, plays an important role in initiating cell proliferation, and is a common indicator of cell proliferation. PCNA protein expression in ovarian tissue was significantly lower in the DHEA group than the control group (Fig. 3). The results confirmed that the follicles in PCOS mice could not develop into mature follicles due to altered proliferation.

### Changes in YAP and p-YAP protein expression in ovarian tissue

**Fig. 5:**
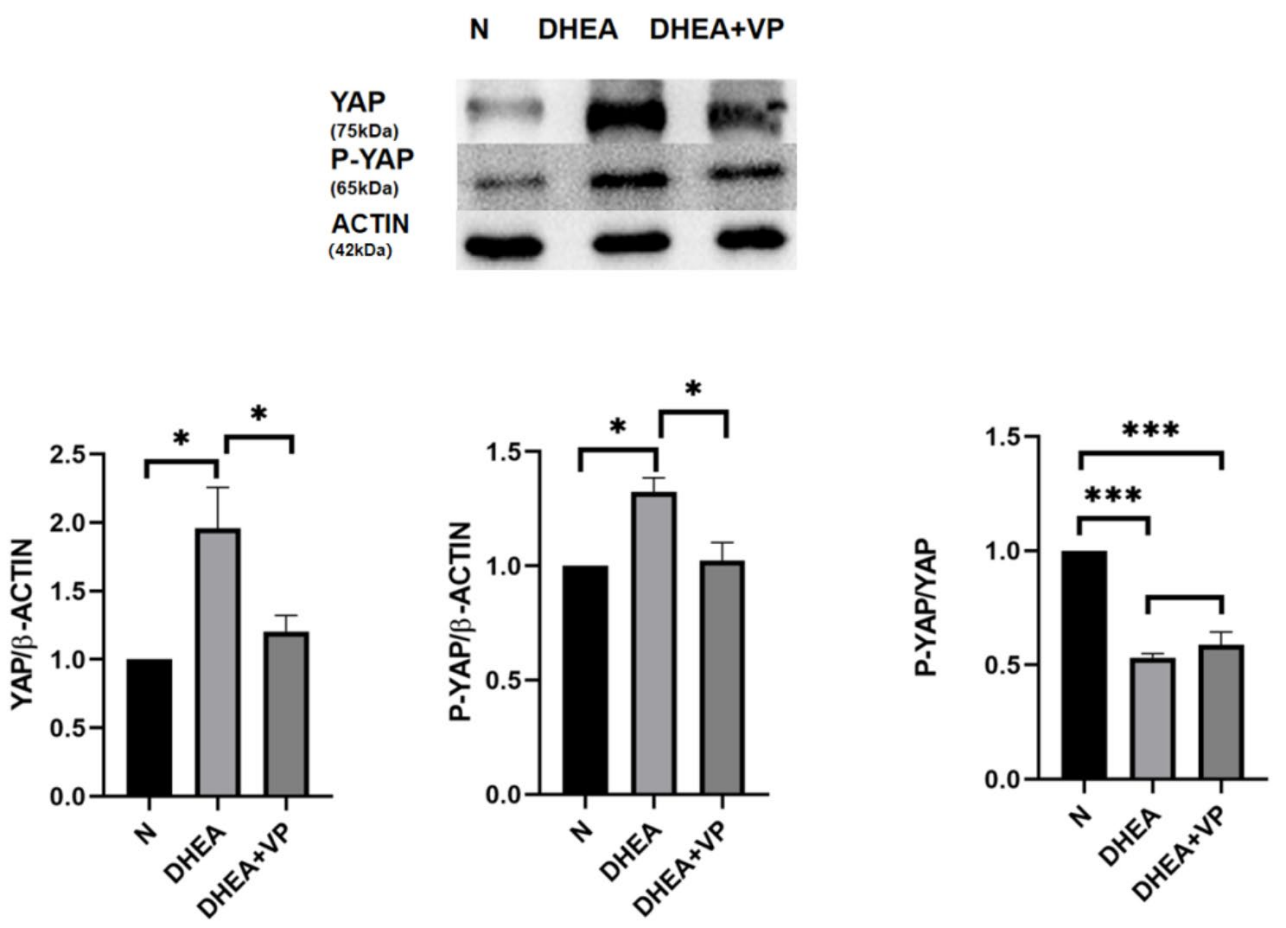
YAP expression, p-YAP expression, and p-YAP/YAP ratio in ovarian tissue in the three groups of mice, assessed by western blotting. N: normal control; DHEA: dehydroepiandrosterone; VP: verteporfin. *P < 0.05, ***P < 0.001

To verify whether DHEA affects the Hippo pathway in ovarian tissue, we first analyzed YAP and p-YAP, which are the key effector proteins in the Hippo pathway. Both YAP and p-YAP protein expression significantly increased after DHEA treatment, but these increases were significantly ameliorated by VP. The p-YAP/YAP ratio was significantly lower in both the DHEA and DHEA + VP groups than the control group (cytoplasmic p-YAP was only slightly increased whereas nuclear YAP was highly increased by DHEA compared to control treatment), causing abnormal proliferation of small antral follicles (Fig. 5). The results confirm that DHEA activates the Hippo pathway in ovaries (though the p-YAP/YAP ratio was lowered) and thereby promotes the proliferation of small antral follicles in ovaries, and Hippo pathway activation is inhibited by VP.

### Changes in MST and LATS1 protein expression in ovarian tissue

**Fig. 6:**
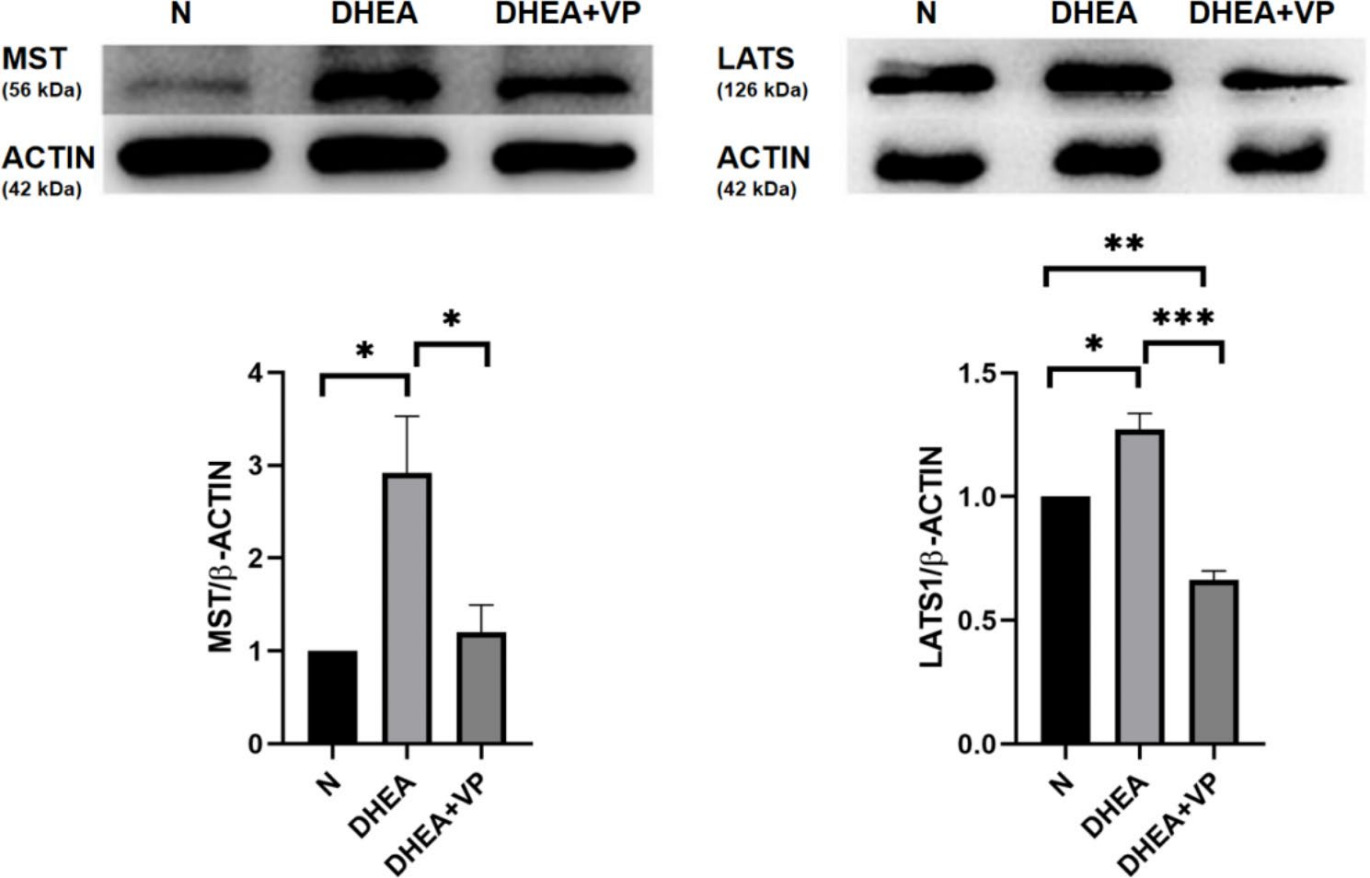
MST and LATS1 protein expression in ovarian tissue in the three groups of mice, assessed by western blotting. N: normal control; DHEA: dehydroepiandrosterone; VP: verteporfin. *P < 0.05, **P < 0.01, ***P < 0.001

To further asses how DHEA affects the ovarian Hippo pathway, we analyzed MST and LATS1 (upstream proteins in the Hippo pathway) in ovarian tissue. MST and LATS1 protein expression increased in the DHEA group, but were significantly decreased by VP (Fig. 6). The results confirmed that the Hippo pathway was activated in ovarian tissue of PCOS mice.

In summary, in the ovarian tissue of DHEA-treated PCOS mice, MST and LATS1 protein expression (upstream proteins in the Hippo pathway, *which promote YAP phosphorylation*) increased and YAP and p-YAP protein expression (downstream proteins in the Hippo pathway) also increased. The large relative increase in (nuclear) YAP may have promoted the proliferation of small antral follicles. The concurrent small relative increase in (cytoplasmic) p-YAP caused the p-YAP/YAP ratio to be decreased in the DHEA group compared to the control group.

## Discussion

PCOS is a common female endocrine disease that can lead to female infertility and seriously affect the physical and mental health of patients. In patients with PCOS, the development of ovarian follicles is disrupted, resulting in a lack of large follicles [4] and an increase in the number of small follicles [5].

The Hippo pathway is related to cell proliferation and growth. Our previous research found that the LATS blocker S1P affected the activation of primordial follicles, changed the proportions of different follicle types in the ovaries, and increased cell proliferation. Research has shown that abnormal follicular development in PCOS is often accompanied by dysregulation of the Hippo pathway. YAP1/TAZ, downstream effectors of the Hippo pathway, are also known to play an important role in granulosa cell proliferation and estradiol synthesis [9, 10]. Additionally, Li et al. showed that there is a difference in YAP protein between patients with PCOS and normal controls [11]. Jiang et al. [12] confirmed YAP methylation abnormalities in PCOS patients. Sun et al. [13] found that Lats1 deletion leads to ovarian germ cell apoptosis and follicular cysts. Therefore, we speculate that the abnormal follicular development in patients with PCOS may be closely related to the Hippo pathway, that is, follicular growth and development may be regulated by the Hippo pathway.

First, we established a PCOS mouse model [14] and administered VP, an inhibitor of YAP (a downstream protein in the Hippo pathway). In the DHEA group compared to the control group, AMH and PCNA in ovarian tissue significantly increased and decreased, respectively; VP significantly ameliorated the increase in AMH but did not ameliorate the decrease in PCNA. This indicated that follicular development was inhibited after the inhibition of YAP by VP, that is, the Hippo pathway affects follicular development [15].

Second, we determined whether there were changes in the expression of Hippo pathway proteins in the ovarian tissue of PCOS mice. The results showed that the levels of YAP and p-YAP (downstream proteins in the Hippo pathway) in ovarian tissue in the DHEA group were significantly increased, indicating abnormalities in the Hippo pathway in the DHEA group. Furthermore, the decrease in the p-YAP/YAP ratio in the DHEA group compared to the control group indicated that nuclear YAP was more highly increased than cytoplasmic p-YAP. The relatively high increase in YAP in the nucleus indicated abnormal cell proliferation in the DHEA group. After administration of VP, p-YAP and YAP protein significantly decreased compared to their levels in the DHEA group, though the p-YAP/YAP ratio did not significantly change.

Third, MST and LATS1 protein expression (upstream proteins in the Hippo pathway) were significantly higher in the DHEA group than the control group, and they were decreased by VP. These results showed that the Hippo pathway was activated in PCOS model mice, which promoted the proliferation of antral follicles. In contrast, the Hippo pathway was inhibited by the YAP inhibitor VP, which decreased the proliferation of antral follicles (as indicated by the decrease in AMH).

To sum up, Hippo pathway activation is closely related to the proliferation of small antral follicles in PCOS. This study provides a theoretical and practical basis for the exploration of the pathogenesis of PCOS and the development of new treatments. Research has shown that there is an association between Hippo pathway activation and iron-dependent cell death [16], and iron-dependent cell death is related to follicular genesis [17]. As the Hippo pathway and iron-dependent cell death are both related to the occurrence of PCOS, there may be a relationship between the Hippo pathway and iron dependent cell death in PCOS and in the changes in ovarian follicles. Further studies on this relationship are needed.

## Data Availability

Data analyzed during this study are included in this published article. Raw data are available from the corresponding author on reasonable request.
