# Correction: Correlation between ovarian follicular development and Hippo pathway in polycystic ovary syndrome

**DOI:** 10.1186/s13048-024-01557-3

**Published:** 2024-11-20

**Authors:** Zichao Huang, Tianyue Xu, Chunling Liu, Honghui Wu, Linglin Weng, Jieyu Cai, Na Liang, Hongshan Ge

**Affiliations:** 1 Graduate School, Nanjing University of Chinese Medicine, Nanjing, China; 2 Reproduction Medicine Centre, The Affiliated Taizhou People’s Hospital of Nanjing Medical University, Taizhou, China; 3 Center for Reproductive Medicine, the Second Affiliated Hospital of Soochow University, Suzhou, Jiangsu 215004 China; 4 Graduate School, Dalian Medical University, Liaoning, China

**Correction: J Ovarian Res 17, 14 (2024)**

10.1186/s13048-023-01305-z

Following publication of the original article [1], the author reported that the internal parameter ACTIN corresponding to AMH in Fig. 2 and the internal parameter ACTIN corresponding to YAP and P-YAP in Fig. 5 have been reused.

**Incorrect figure**


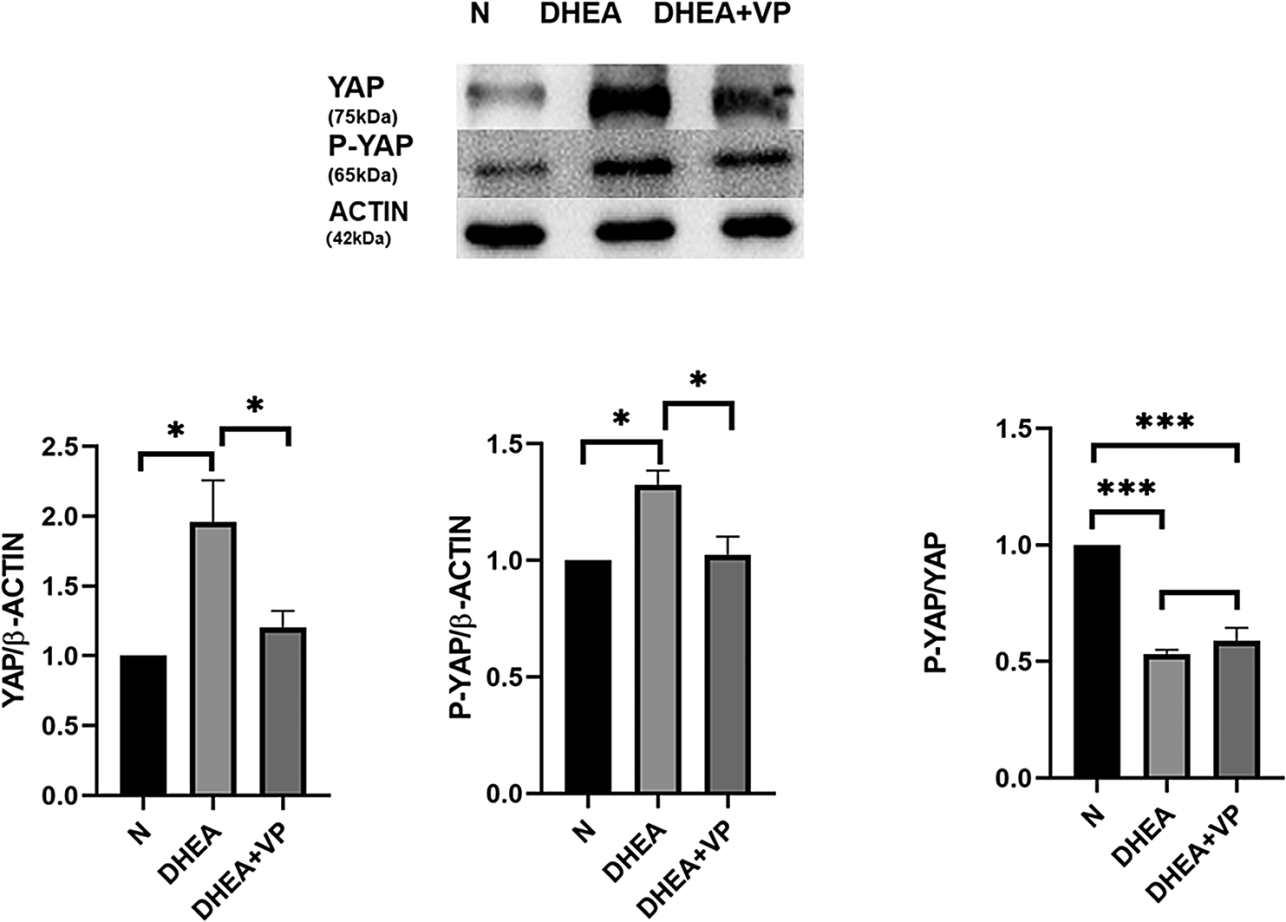


**Correct figure 5**


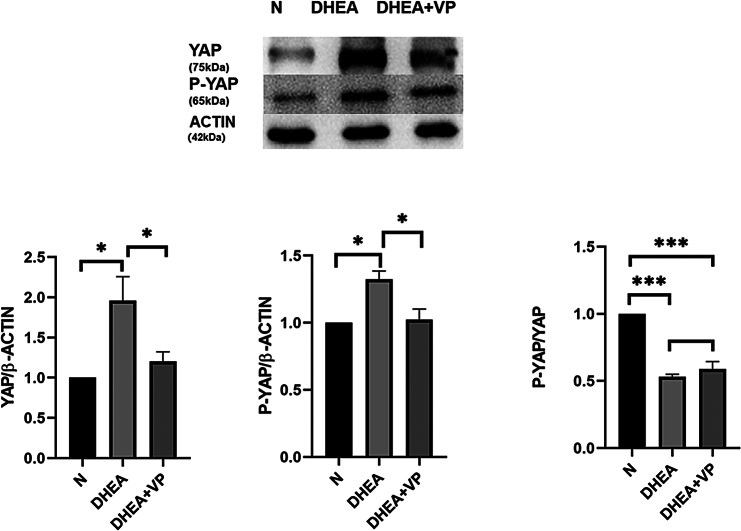


The original article [1] has been corrected.
